# Readmissions and Mortality During the First Year After Stroke—Data From a Population-Based Incidence Study

**DOI:** 10.3389/fneur.2020.00636

**Published:** 2020-07-24

**Authors:** Pedro Abreu, Rui Magalhães, Diana Baptista, Elsa Azevedo, Maria Carolina Silva, Manuel Correia

**Affiliations:** ^1^Department of Neurology, Centro Hospitalar Universitário de São João, Porto, Portugal; ^2^Department of Clinical Neurosciences and Mental Health, Faculdade de Medicina, Universidade Do Porto, Porto, Portugal; ^3^Instituto de Ciências Biomédicas Abel Salazar, Universidade Do Porto, Porto, Portugal; ^4^Department of Neurology, Hospital Santo António—Centro Hospitalar Universitário Do Porto, Porto, Portugal

**Keywords:** stroke readmissions, epidemiology, outcome, mortality, community-based study

## Abstract

**Background:** After a first-ever-in-a-lifetime stroke (FELS), hospital readmissions are common and associated with increased mortality and morbidity of stroke survivors, thus, raising the overall health burden of stroke. Population-based stroke studies on hospital readmissions are scarce despite it being an important healthcare service quality indicator. We evaluated unplanned readmissions or death during the first year after a FELS and their potential factors, based on a Portuguese community register.

**Methods:** Data were retrieved from a population-based prospective register undertaken in Northern Portugal (ACIN2) in 2009–2011. Retrospective information about unplanned hospital readmissions and case fatality within 1 year after FELS index hospitalization (FELS-IH) was evaluated. Readmission/death-free survival 1 year after discharge was estimated using the Kaplan–Meyer method. Independent risk factors for readmission/death were identified using Cox proportional hazard models.

**Results:** Unplanned readmission/death within 1 year occurred in 120 (31.6%) of the 389 hospitalized FELS survivors. In 31.2% and 33.5% of the cases, it occurred after ischemic stroke or intracerebral hemorrhage, respectively. Infections and cerebrovascular and cardiovascular diseases were the main causes of readmission. Of the readmissions, 65.3% and 52.5% were potentially avoidable or stroke related, respectively. The main cause of potentially avoidable readmissions was the continuation/recurrence of the event responsible for the initial admission or a closely related condition (71.2%). Male sex, age, previous and post-stroke functional status, and FELS-IH length of stay were independent factors of readmission/death within 1 year.

**Conclusions:** Almost one-third of FELS survivors were readmitted/dead 1 year after their FELS-IH. This outcome persisted after the first months after stroke hospitalization in all stroke subtypes. More than half of readmissions were considered potentially avoidable or stroke related.

## Introduction

After a first-ever-in-a-lifetime stroke (FELS) or transient ischemic attack (TIA), the use of hospital emergency services or hospital readmissions is common and associated with increased stroke mortality and morbidity, thus, raising the overall health burden of stroke (1). Also, despite some well-characterized limitations (2), readmissions are currently a measure of the hospital's performance and quality of care (3).

Several risk factors for stroke readmissions have been described. However, many meaningful clinical associations may have been ignored since most studies only rely on large administrative or single-hospital databases, particular subtypes of stroke, or readmissions in the first 3 months after stroke (1, 4, 5). This assertion is especially true in Portugal, where, to our knowledge, there are no population-based stroke readmission studies, and therefore, the corresponding information is scarce.

We aimed to study unplanned readmissions or death during the first year after a FELS and to identify their potential factors, based on a Portuguese community register.

## Materials and Methods

The sample was obtained from the second population-based register undertaken in Northern Portugal (ACIN2), comprising all FELS recorded between October 2009 and September 2011 in the population registered in the Health Centers Group of Western Porto main city (190,000 persons) and two health centers in rural regions in Northern Portugal (Mirandela and Vila Pouca de Aguiar, involving about 46,000 persons) (6). Multiple sources of information were used to identify all patients with a FELS using a record-linkage methodology based on the National Health Number, a unique identifier for residents in Portugal to contact the National Health Service (NHS). Hot-pursuit and cold-pursuit ascertainment involving community-based and hospital-based information sources were used (6). Hot-pursuit encompassed a daily review of emergency admissions and referrals to the project out-patient clinic at Hospital de Santo António. Cold pursuit was used to check for completeness of hot-pursuit identification (7). Patients were examined as soon as possible after symptoms' onset at the emergency room, during their hospital stay or at the project out-patient clinic, within 1 month and then were followed-up until 3 months after a stroke. More detailed information is described elsewhere (6). This study includes all patients from Porto admitted to the hospital after a FELS. The information about readmissions after the 3-months follow-up period was collected retrospectively. The ethics committee of the Centro Hospitalar Universitário de São João and the Centro Hospitalar Universitário do Porto approved this study.

The World Health Organization's “stroke” definition and Sudlow's and Warlow's stroke pathological types—ischemic stroke (IS), intracerebral hemorrhage (ICH), and subarachnoid hemorrhage (SAH)—were considered for the corresponding concepts (8, 9). Brain image (computerized tomography scan/magnetic resonance imaging) was used to confirm stroke types. The TOAST criteria were used to define IS etiology and the Bamford Oxfordshire classification to define clinical IS syndromes (10, 11).

Stroke severity at the first medical evaluation was characterized as mild, moderate, or severe based on the National Institutes of Health Stroke Scale (NIHSS) (12) (NIHSS ≤7, 8–16, or ≥17, respectively), except for SAH. Whenever the NIHSS was unavailable, the score was estimated retrospectively from the patients' clinical records, if valid for that purpose (13). The pre and post-stroke (~28 days after stroke) functional outcome was assessed with the modified Rankin Scale (mRS) (14).

The following criteria were considered as pre-stroke risk factors: (a) history of hypertension or antihypertensive treatment; (b) previous diagnosis/treatment of diabetes mellitus with oral antidiabetic agent/insulin or fasting glycemia >126 mg/dl, postprandial glycemia ≥200 mg/dl, and/or ≥200 mg/dl in the 2-h glucose tolerance test; (c) evidence of atrial fibrillation in electrocardiogram or documented in the patient's records; (d) previous diagnosis/treatment of hypercholesterolemia; (e) history of myocardial infarction; (f) current smoking habits (if patients had smoked at all in the preceding 12 months) (6). Other pre-stroke comorbidities such as congestive heart failure, dementia, HIV infection, and malignant neoplasm were included after reviewing patients' medical records using the International Classification of Diseases 9th Revision (ICD-9) diagnosis code.

Planned readmissions were defined as readmissions to perform a scheduled procedure (e.g., carotid endarterectomy or stenting, patent *foramen ovale* closure, cardiac planned procedures, and cranioplasty) and planned hospitalizations (rehabilitation, chemo- or radiotherapy treatment, major organ transplant, or obstetrical delivery) (15).

Unplanned readmissions were defined as >24-h hospitalizations due to unexpected causes and emergency episodes leading to death that did not fulfill any planned readmission criterion that had occurred within 1 year of the FELS index hospitalization (FELS-IH) (15). Unplanned readmissions after a planned admission were also acknowledged.

Patients who died during their FELS-IH were excluded. Two neurology study investigators (including a stroke neurologist) reviewed the patients' medical records using the ICD-9 diagnosis code to obtain and validate the unplanned readmission causes. The main unplanned readmission diagnosis-related group code was identified for statistical data and subgroup analyses. A composite outcome event of unplanned first-ever readmission or death without readmission within 1 year after FELS was considered to capture all negative health outcomes (3).

Potentially avoidable readmissions (PAR) were defined as causes that could have been prevented or modified during the FELS-IH, and their clinical plausibility was defined using Goldfield et al.'s criteria (16): (a) medical readmission for a continuation or recurrence of the reason for the initial admission, or for a closely related condition; (b) medical readmission for an acute decompensation of a chronic problem that was not the reason for the initial admission, but was plausibly related to care either during or immediately after the initial admission; (c) medical readmission for an acute medical complication plausibly related to care during the initial admission; (d) readmission for a surgical procedure to address a continuation or a recurrence of the problem causing the initial admission; (e) readmission for a surgical procedure to address a complication resulting from care during the initial admission. In case of disagreement, the study investigators reached a consensus for the readmission classification.

Stroke-related readmissions were defined as recurrent vascular events and complications that warranted readmission, including stroke, pneumonia, urinary tract infection, peripheral and coronary artery disease, hip fracture, and pulmonary embolism (17, 18).

### Statistics

Sociodemographic characteristics were summarized using descriptive statistics. The baseline and clinical characteristics of readmitted vs. non-readmitted patients were compared using the Chi-square or the Fisher exact test when adequate for categorical variables and the *t*-test or the Mann–Whitney *U* test for continuous variables (normality of distributions was assessed using the Shapiro–Wilk test). The overall cumulative readmission/death-free survival and PAR-free survival over 12 months was estimated using the Kaplan–Meyer method. Independent risk factors for readmission were evaluated using Cox proportional hazard models. ICH and SAH were combined as hemorrhagic stroke (HS) for the Kaplan–Meyer survival estimation (Figure 2) and the description of characteristics and reasons for all-cause readmissions by sub-groups (Table 3). A value of *p* = 0.05 was considered as the limit to wrongly reject the null hypothesis. Data analysis was performed using SPSS Statistics v24.

## Results

### Study Cohort

**Figure 1:**
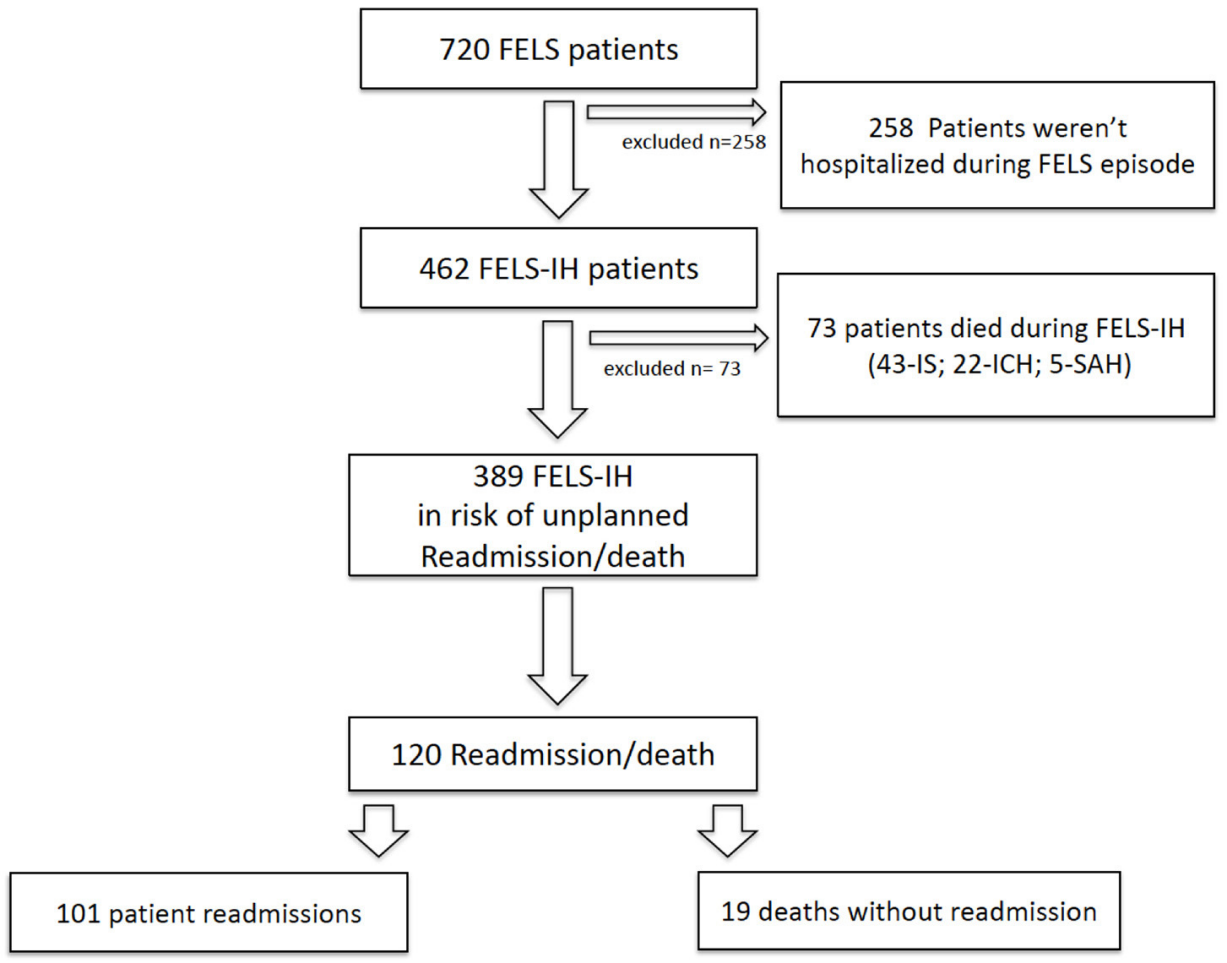
Study design for cohort follow-up. FELS, first-ever-in-a-lifetime stroke; FELS-IH, first-ever-in-a-lifetime-stroke index hospitalization; IS, ischemic stroke, ICH, intracerebral hemorrhage; SAH, subarachnoid hemorrhage.

Figure 1 shows the study design for the cohort follow-up. From the initial cohort of 720 FELS patients in the ACIN2 database, we excluded 258 not hospitalized in the index event and 73 that died during the FELS-IH; 389 FELS patients at risk of an unplanned readmission/death were included. This cohort had a mean age of 70 years and 208 (53.5%) women; 317 (81.5%) had an IS in the index event, 58 (14.9%) an ICH, and 14 (3.6%) an SAH. All patients had a brain image performed.

Table 1 shows the baseline characteristics of non-readmitted and readmitted/deceased patients. Readmitted/deceased patients were older (77.1 vs. 67.1, *p* < 0.001), had higher pre-stroke dependency (mRS ≥ 2, 50.8% vs. 25.7%, *p* < 0.001), and had more often hypertension (83.3% vs. 73.2%, *p* = 0.03), atrial fibrillation (AF; 32.5% vs. 20.1%, *p* = 0.008), and congestive heart failure (25.0% vs. 16.4%, *p* = 0.045). They also had had more total anterior circulation infarcts and less lacunar and posterior circulation infarcts (*p* = 0.008), more cardioembolic strokes and less small vessels or other determined infarcts (*p* = 0.019). Moreover, readmitted/deceased patients had a higher NIHSS score (9 vs. 4, *p* < 0.001), more moderate or severe strokes (*p* < 0.001), higher post-stroke dependency levels (mRS ≥ 2, 95.0% vs. 71.4%, *p* < 0.001), higher likelihood of having had a previous hospital admission (18.3% vs. 8.9%, *p* = 0.008), and a higher median in-hospital length of stay (LoS) (11 vs. 8 days, *p* = 0.008). We found no differences between non-readmitted and readmitted/deceased patients regarding other baseline characteristics.

**Table 1:** Patients' characteristics at baseline (non-readmitted and readmitted/death).

	**Overall (*****n*** **=** **389)**		**Non-readmitted (*****n*** **=** **269)**		**Readmitted/death (*****n*** **=** **120)**		
	***n***	**%**	***n***	**%**	***n***	**%**	***p*-value**
Women	208	53.5	142	52.8	66	55.0	0.686
Mean age (SD), years	70.2	(15.1)	67.1	(15.3)	77.1	(12.2)	**<0.001**^b^
<45	29	7.5	27	10.0	2	1.7	**<0.001**
45–64	100	25.7	79	29.4	21	17.5	
65–84	195	50.1	140	52.0	55	45.8	
≥85	65	16.7	23	8.6	42	35.0	
Pre-stroke mRS≥2	130	33.4	69	25.7	61	50.8	**<0.001**
Pre-existing comorbidities							
Hypertension	297	76.3	197	73.2	100	83.3	**0.030**
Diabetes mellitus	102	26.2	68	25.3	34	28.3	0.527
Atrial fibrillation	93	23.9	54	20.1	39	32.5	**0.008**
Myocardial infarction	39	10.0	28	10.4	11	9.2	0.706
Hypercholesterolemia	182	46.8	134	49.8	48	40.0	0.073
Smoking	144	37.0	108	40.1	36	30.0	0.056
Congestive heart failure	74	19.0	44	16.4	30	25.0	**0.045**
Dementia	45	11.6	28	10.4	17	14.2	0.285
Neoplasm	60	15.4	36	13.4	24	20.0	0.095
HIV	6	1.5	5	1.9	1	0.8	0.448
Stroke pathological type							0.802
Ischemic stroke	317	81.5	221	82.2	96	80.0	
Intracerebral hemorrhage	58	14.9	38	14.1	20	16.7	
Subarachnoid hemorrhage	14	3.6	10	3.7	4	3.3	
Ischemic stroke subtype							**0.008**
Total anterior circulation infarct	79	24.9	44	19.9	35	36.5	
Partial anterior circulation infarct	102	32.2	71	32.1	31	32.3	
Lacunar infarct	62	19.6	48	21.7	14	14.6	
Posterior circulation infarct	74	23.3	58	26.2	16	16.7	
Ischemic stroke etiology							**0.019**
Large-artery atherosclerosis	49	15.5	34	15.4	15	15.6	
Cardioembolism	103	32.5	64	29.9	39	40.6	
Small-artery occlusion	51	16.1	41	18.6	10	10.4	
Other determined	14	4.4	14	6.3	0	0.0	
Undetermined	100	31.5	68	30.8	32	33.3	
Median NIHSS (IQR)^a^	5	(2–13)	4	(2–11)	9	(4–14)	**<0.001**^c^
Stroke severity (NIHSS)^a^							**<0.001**
≤ 7	229	61.1	175	67.6	54	46.6	
8–16	92	24.5	49	18.9	43	37.1	
≥17	54	14.4	35	13.5	19	16.4	
Post-stroke mRS≥2	306	78.7	192	71.4	114	95.0	**<0.001**
Management							
Delay less than 3 h	199	51.2	144	53.5	55	45.8	0.161
Thrombolysis	48	15.1	33	14.9	15	15.6	0.874
Stroke occurred while in hospital	29	7.5	20	7.4	9	7.5	0.982
Median hospital length of stay (IQR), days	9	(4–19)	8	(4–18)	11	(5–27)	**0.022**^c^
Previous admissions	46	11.8	24	8.9	22	18.3	**0.008**

*SD, standard deviation; mRS, modified Rankin Scale; IQR, interquartile range*.

a *National Institutes of Health Stroke Scale (NIHSS) excluding subarachnoid hemorrhages*.

b *t-test*.

c *Mann–Whitney U test*.

*Bold entries indicate statistical significance (p < 0.05)*.

### Readmission or Death Rates

**Figure 2:**
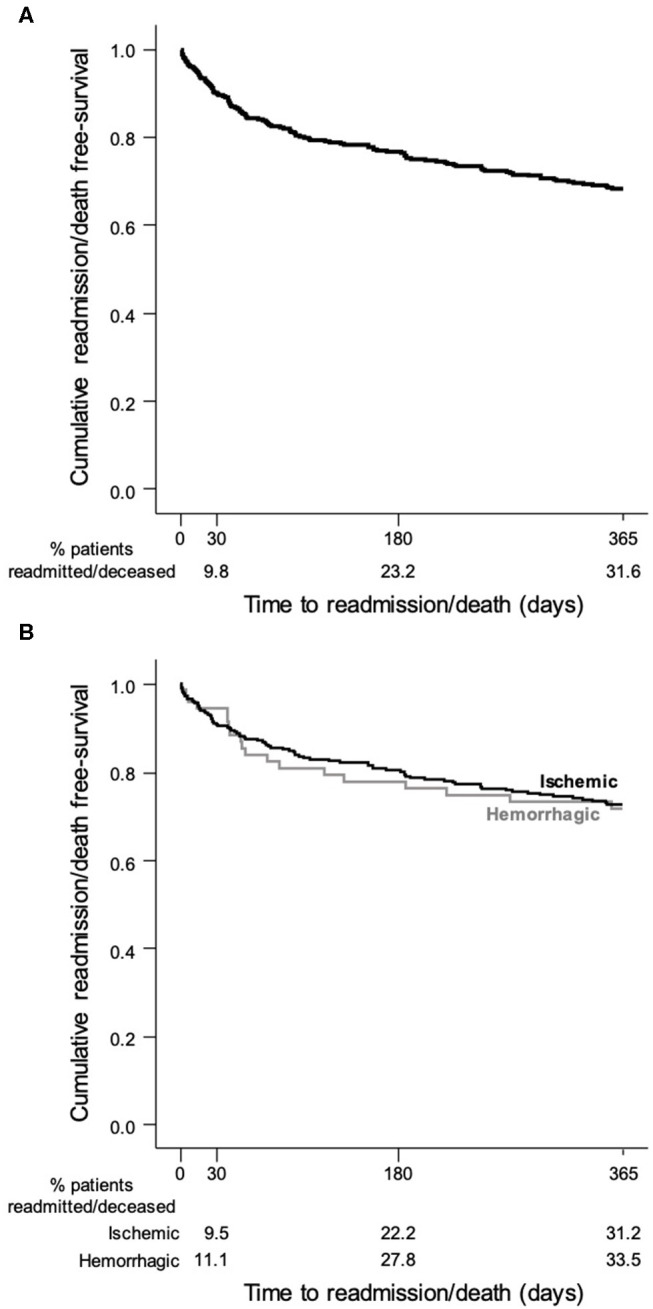
Kaplan–Meier survival curve showing the probability of a stroke patient will remain free of readmission/death after discharge: **(A)** all stroke patients; **(B)** stroke patients stratified by pathological type.

Figure 2 shows the cumulative readmission or death rates. The all-cause readmission/death rate was 9.8, 23.2, and 31.6% at 30 days, 180 days, and 1 year, respectively. In cases of IS and HS, the rate was, respectively, 9.5 and 11.1% at 30 days, 22.2% and 27.8% at 180 days, and 31.2% and 33.5% at 1 year.

### Overall Unplanned Readmission Rates and Mortality Without Readmission

Overall, 120 patients were readmitted/deceased within 1 year: 101 (84.2%) unplanned readmissions and 19 (15.8%) deaths without readmission (Figure 1). Cumulative rates of readmission were similar to the readmission/death rates (data not shown). Of the 19 patients who had deceased without readmission at 1 year, 1/19 died within 30 days, 12/19 within 30–180 days, and 6/19 within 180–365 days.

### Readmissions Causes and Characterization

Tables 2–4 summarize the patients' characteristics and the reasons for the hospital readmissions within the first year. The three most common reasons for readmission were infectious diseases (39.6%), cerebrovascular diseases (15.8%)—particularly IS (9.9%), and cardiovascular diseases (8.9%). The median time for readmission after FELS-IH was 59 days (interquartile range (IQR): 23–183 days). Of the unplanned readmissions, 32.7% occurred within 30 days, 41.6% within 30–180 days, and 25.7% within 180–365 days. The median in-hospital LoS of readmissions was 7 days (IQR: 3–17 days). Nineteen patients (18.8%) died during readmission: 7/19 within 30 days, 9/19 within 30–180 days, and 3/19 within 180–365 days.

**Table 2:** Characteristics and causes of patients' all-cause readmissions within 1 year.

		**Days from the first-ever readmission**						
	**Overall** ***n*** **=** **101**		**<30 days** ***n*** **=** **33**		**30–180 days** ***n*** **=** **42**		**180–365 days** ***n*** **=** **26**	
	***n***	**%**	***n***	**%**	***n***	**%**	***n***	**%**
Median hospital stays (IQR), days	7	(3–17)	9	(3–23)	7	(3–17)	8	(3–14)
Readmission case fatality^a^	19	18.8	7	21.2	9	20.1	3	11.5
**Causes of unplanned readmissions**								
Infectious diseases	40	39.6	13	39.4	19	45.2	8	30.8
Respiratory tract	17	42.5	4	30.7	8	42.1	5	62.5
Urinary tract	12	30.0	6	46.2	6	31.6	0	0.0
Sepsis	7	17.5	2	15.4	3	15.7	2	25
Other	4	10.0	1	7.7	2	10.5	1	12.5
Cerebrovascular disease (IS)	16 (10)	15.8 (9.9)	6 (5)	18.2 (15.2)	7 (4)	16.7 (9.5)	3 (1)	11.5 (3.8)
Cardiovascular disease^b^ (MI)	9 (3)	8.9 (3.0)	2 (0)	6.1 (0.0)	4 (1)	9.5 (2.4)	3 (2)	11.5 (7.7)
Neoplasm	8	7.9	3	9.1	1	2.4	4	15.4
Gastrointestinal diseases (esophagitis/gastritis)	5 (3)	5.0 (3.0)	1 (1)	3.0 (3.0)	2 (1)	4.8 (2.4)	2 (1)	7.7 (3.8)
Chronic respiratory diseases	4	4.0	0	0.0	2	4.8	2	7.7
Other	19	18.8	8	24.2	7	16.7	4	15.4
Potentially avoidable readmission^c^	66	65.3	23	69.7	32	76.2	11	42.3
Stroke-related readmission^d^	53	52.5	16	48.5	26	61.9	11	42.3

*IS, ischemic stroke; MI, myocardial infarction; IQR, interquartile range*.

a *Readmission cases*.

b *Cardiovascular disease includes myocardial infarction, arrhythmia, congestive heart failure, and valvular disease*.

c *Potentially avoidable readmissions include medical readmission for a continuation or recurrence of the reason for the initial admission, or a closely related condition; medical readmission for an acute decompensation of a chronic problem that was not the reason for the initial admission, but was plausibly related to care either during or immediately after the initial admission; medical readmission for an acute medical complication plausibly related to care during the initial admission*.

d *Stroke-related readmissions include recurrent vascular events and complications that warranted readmission, including stroke, pneumonia, urinary tract infection, peripheral and coronary artery disease, hip fracture, and pulmonary embolism*.

**Table 3:** Characteristics and causes of patients' all-cause readmissions within 1 year, by sub-groups: stroke type and age.

	**Ischemic stroke (*****n*** **=** **82)**				**Hemorrhagic stroke (*****n*** **=** **19)**^****a****^			
	** <65 years old**		**≥65 years old**		** <65 years old**		**≥65 years old**	
	***n***	**%**	***n***	**%**	***n***	**%**	***n***	**%**
Unplanned readmissions—all causes	14	17.1	68	82.9	5	26.3	14	73.7
Infectious diseases	-	-	31	45.6	2	40.0	7	50.0
Respiratory tract	-	-	16	51.6	-	-	1	14.3
Urinary tract	-	-	9	29	1	50.0	2	28.6
Sepsis	-	-	3	9.7	-	-	4	57.1
Other	-	-	3	9.7	1	50.0	-	-
Cerebrovascular disease (ischemic stroke)	4 (3)	4.9 (3.7)	8 (6)	11.7(8.8)	-	-	4 (1)	28.6 (7.1)
Cardiovascular disease^b^ (myocardial infarction)	2 (0)	2.4 (0.0)	5 (2)	6.1 (2.9)	1 (1)	20.0 (20.0)	1 (0)	7.1 (0.0)
Neoplasm	2	14.3	5	7.4	-	-	-	-
Gastrointestinal diseases (esophagitis/gastritis)	1 (1)	7.1 (7.1)	3 (1)	4.4 (1.5)	-	-	-	-
Chronic respiratory diseases	2	14.3	1	1.5	-	-	1	7.1
Other	3	2.1	15	22.1	2	40.0	1	7.1
Potentially avoidable readmissions^c^	7	50.0	43	63.2	2	40.0	14	100.0
Stroke-related readmissions^d^	4	28.6	40	58.8	2	40.0	7	50.0
Readmission case-fatality	1	7.1	16	23.5	-	-	2	14.3

a *Includes intracerebral and subarachnoid hemorrhage*.

b *Cardiovascular disease includes myocardial infarction, arrhythmia, congestive heart failure and valvular disease*.

c *Potentially avoidable readmissions include medical readmission for a continuation or recurrence of the reason for the initial admission or a closely related condition; medical readmission for an acute decompensation of a chronic problem that was not the reason for the initial admission but was plausibly related to care either during or immediately after the initial admission; medical readmission for an acute medical complication plausibly related to care during the initial admission*.

d *Stroke-related readmissions include recurrent vascular events and complications that warranted readmission, including stroke, pneumonia, urinary tract infection, peripheral and coronary artery disease, hip fracture, and pulmonary embolism*.

**Table 4:** Baseline characteristics of patients' readmitted within 1 year, by sub-groups: avoidable and non-avoidable.

	**Overall (*****n*** **=** **101)**		**Non-avoidable (*****n*** **=** **35)**		**Avoidable (*****n*** **=** **66)**		
	***n***	**%**	***n***	**%**	***n***	**%**	***p-*value**
Women	55	54.5	17	48.6	38	57.6	0.387
Mean age (SD), years	76.9	(11.9)	73.3	(13.3)	78.7	(10.7)	**0.035**^b^
<45	2	7.5	2	5.7	0	0.0	
45–64	17	25.7	8	22.9	9	13.6	
65–84	49	50.1	18	51.4	31	47	
≥85	33	16.7	7	20.0	26	39.4	
Pre-stroke mRS ≥ 2	51	50.5	16	45.7	35	53	0.484
Pre-existing comorbidities							
Hypertension	84	83.2	29	82.9	55	83.3	0.951
Diabetes mellitus	27	26.7	11	31.4	16	24.2	0.437
Atrial fibrillation	32	31.7	9	25.7	23	34.8	0.348
Myocardial infarction	9	8.9	1	2.9	8	12.1	0.120
Hypercholesterolemia	43	42.6	16	45.7	27	40.9	0.642
Smoking	32	31.7	12	34.3	20	30.3	0.682
Congestive heart failure	25	24.8	6	17.1	19	28.8	0.197
Dementia	12	11.9	2	5.7	10	15.2	0.290
Neoplasm	19	18.8	11	31.4	8	12.1	**0.018**
HIV	1	1.0	1	2.9	0	0.0	0.347
Stroke pathological type							0.216
Ischemic stroke	82	81.2	32	91.4	50	75.8	
Intracerebral hemorrhage	15	14.9	3	8.6	12	18.2	
Subarachnoid hemorrhage	4	3.9	0	0.0	4	6.1	
Ischemic stroke subtype							0.129
Total anterior circulation infarct	30	36.6	7	21.9	23	46.0	
Partial anterior circulation infarct	24	29.3	10	31.3	14	28.0	
Lacunar infarct	13	15.9	7	21.9	6	12.0	
Posterior circulation infarct	15	18.3	8	25.0	7	14.0	
Ischemic stroke etiology							0.702
Large-artery atherosclerosis	12	14.6	7	21,9	5	10.0	
Cardioembolism	36	43.9	11	34.4	25	50.0	
Small-artery occlusion	10	12.2	5	15.6	5	10.0	
Other determined	5	6.1	3	9.4	2	4.0	
Undetermined	19	23.2	8	25.0	11	22.0	
Median NIHSS (IQR)^a^	8	(4–14)	6	(3–9)	10	(5–15)	**0.008**^c^
Stroke severity (NIHSS)^a^							**0.005**
≤ 7	48	49.5	25	71.4	23	37.1	
8–16	35	36.1	7	20.0	28	45.2	
≥17	14	14.4	3	8.6	11	17.7	
Post-stroke mRS ≥ 2	95	94.1	33	94.3	62	93.9	0.657
Management							
Delay less than 3 h	46	45.5	15	42.9	31	47.0	0.693
Thrombolysis	14	13.9	4	11.4	10	15.2	0.606
Stroke occurred while in hospital	7	6.9	3	8.6	4	6.1	0.636
Median hospital length of stay (IQR), days	10	(5–22)	8	(3–18)	12.5	(7–26)	**0.030**^c^
Previous admissions	18	17.8	9	25.7	9	13.6	0.257

*SD, standard deviation; mRS, modified Rankin Scale; IQR, interquartile range*.

a *National Institutes of Health Stroke Scale (NIHSS) excluding subarachnoid hemorrhages*.

b *t-test*.

c *Mann–Whitney U test*.

*Bold entries indicate statistical significance (p < 0.05)*.

### Potentially Avoidable Readmissions

**Figure 3:**
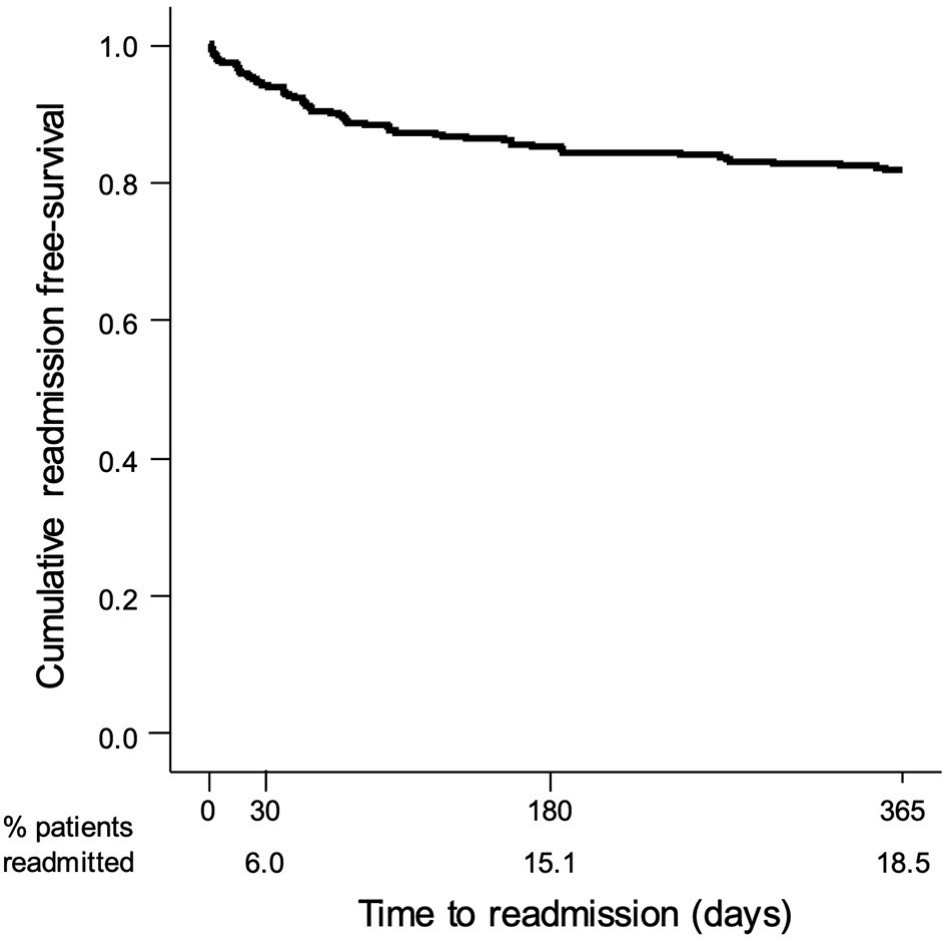
Kaplan–Meier survival curve showing the probability of a stroke patient remaining free of non-avoidable readmissions after discharge.

PAR occurred in 66 (65.3%) of the readmitted patients: 47 (71.2%) due to medical readmission for a continuation or recurrence of the reason for the initial admission, or for a closely related condition; 6 (9.1%) due to medical readmission for an acute decompensation of a chronic problem that was not the reason for the initial admission but was plausibly related to care either during or immediately after the initial admission; and 13 (19.7%) due to a medical readmission for an acute medical complication plausibly related to care during the initial admission; and no readmissions for a surgical procedure were observed. The PAR rate was 6.0, 15.1, and 18.5% at 30 days, 180 days, and 1 year, respectively (Figure 3). Compared to non-avoidable readmissions (Table 4), PAR patients were older (78.7 vs. 73.3 years old, *p* = 0.035), had less neoplasms (31.4% vs. 12.1%, *p* = 0.018), a higher NIHSS score (10 vs. 6, *p* = 0.008), more moderate or severe strokes (*p* = 0.008), and a higher median in-hospital LoS (13 vs. 8 days, *p* = 0.030).

### Stroke-Related Readmissions

Stroke-related readmissions represented 52.5% of readmissions and occurred in 48.5, 61.9, and 42.3% of the patients readmitted within 30 days, 30–180 days, and 180–365 days, respectively (Table 2).

### Factors Associated With Readmission or Death

Table 5 shows the univariate and multivariate analyses for all-cause readmission or death within 1 year. Age, previous functional status, hypertension, AF, congestive heart failure, initial NIHSS score, post-stroke functional status, disabling stroke, FELS-IH LoS, and previous admissions were significantly associated with readmission or death within 1 year, in the univariate model analysis. Conversely, being a current smoker was negatively associated with this outcome. In the multivariate regression analysis, only the male gender, age, previous and post-stroke functional status, and FELS-IH LoS remained independent factors of readmission/death within 1 year.

**Table 5:** Univariate and multivariate readmission analyses.

	**Univariate**			**Multivariate**		
	**HR**	**95%CI**	***p*-value**	**HR**	**95%CI**	***p*-value**
Men vs. women	0.88	0.61–1.26	0.478	2.09	1.21–3.60	**0.008**
Age, years	1.05	1.04–1.07	** <0.001**	1.04	1.02–1.07	** <0.001**
Pre-stroke mRS ≥ 2 vs. mRS <2	2.59	1.81–3.70	** <0.001**	1.77	1.20–2.63	**0.004**
Pre-existing comorbidities (yes vs. no)						
Hypertension	1.73	1.07–2.80	**0.025**	1.25	0.74–2.11	0.398
Diabetes mellitus	1.14	0.77–1.70	0.506	1.03	0.67–1.59	0.906
Atrial fibrillation	1.76	1.20–2.58	**0.004**	1.06	0.69–1.62	0.807
Myocardial infarction	0.88	0.47–1.64	0.689	0.61	0.31–1.19	0.146
Hypercholesterolemia	0.73	0.51–1.06	0.094	0.81	0.55–1.19	0.809
Smoking	0.64	0.43–0.94	**0.023**	0.58	0.33–1.00	0.051
Congestive heart failure	1.60	1.06–2.41	**0.027**	1.00	0.64–1.57	0.992
Dementia	1.41	0.85–2.36	0.186	0.80	0.46–1.37	0.412
Neoplasm	1.43	0.92–2.24	0.114	1.36	0.85–2.18	0.196
HIV	0.45	0.06–3.21	0.425	0.94	0.12–7.18	0.948
Ischemic stroke vs. others	0.88	0.57–1.38	0.589	0.81	0.51–1.29	0.372
NIHSS^*^	1.04	1.01–1.06	**0.002**			
Post-stroke mRS ≥ 2	6.38	2.81–14.5	** <0.001**	3.17	1.34–7.49	**0.009**
Hospital stay, days	1.01	1.00–1.01	**0.003**	1.01	1.00–1.01	**0.046**
Previous admissions	1.96	1.23–3.11	**0.004**	1.32	0.80–2.19	0.273

*mRS, modified Rankin Scale; NIHSS, National Institutes of Health Stroke Scale*.

* *NIHSS was calculated only for ischemic and hemorrhagic strokes*.

*Bold entries indicate statistical significance (p < 0.05)*.

## Discussion

Our study showed that 31.6% of FELS-IH survivors were readmitted/dead within 1 year. Infections, stroke recurrence, and cardiovascular diseases were the most common causes. More than half of the readmissions were PAR or stroke-related. Only the male gender, age, previous and post-stroke functional status, and FELS-IH LoS were independent factors of readmission/death.

In literature, the 1-year stroke readmission/death rate varies between 13 and 62% (4, 5, 19). Such a wide variation reflects different study methodologies and national health realities. Our results are within this range. Likewise, our readmission/death rate by stroke type confirms the lower-end range of previously reported readmission/death rates regarding IS (31 to 49%) (20, 21) and ICH (33 to 44.7%) (22–24), particularly in two Portuguese single-data center studies (IS, 34%; ICH, 33%) (22, 25).

The main differences between non-readmitted and readmitted/deceased patients (e.g., age, stroke comorbidities, stroke type/etiology, pre- and post-stroke disability, LoS, and previous admissions) fit the pattern of other readmission studies' cohorts, such as those identified by Koennecke et al., of in-hospital worse stroke outcome/morbidity (26). Hence, these factors may help recognize individuals who are more vulnerable to readmission and must be considered in the clinical setting (1, 4, 26, 27). One-third of readmissions/deaths in our cohort occurred within 30 days after discharge. Nevertheless, the readmission/death risk endured after this period, as reported in other studies (18). Also, readmission case fatality at 30 days was higher than that reported for the general all-hospital in a Portuguese readmission administrative database study (21 vs. 9.5%) (28). As in the French Dijon Stroke registry cohort, this finding shows that readmission negatively affected survival (29).

In our study, the main causes of readmission were infections, stroke recurrence, and cardiovascular diseases, as in other cohorts (1). While infectious diseases dominated the 1-year readmission causes, over the year, readmissions due to cerebrovascular diseases decreased, and those due to cardiovascular diseases increased; this suggests an increasing importance of cardiac disease overtime after stroke (21, 30).

More than half of our readmissions were PAR, and these were highest within 180 days after FELS-IH. The few reports addressing this issue in stroke cohorts study PAR only within a 30-days period, and comparatively, PAR at 30 days were higher in our study (31–34). Nonetheless, this difference may reflect a different methodology for PAR report more than a contrasting stroke treatment reality (34). Moreover, we identified differences in the characteristics of PAR patients compared to non-avoidable readmission patients that may explain their proneness to this type of readmission; e.g., they were older, had higher NIHSS scores, more severe strokes, and higher median in-hospital LoS.

Theoretically, most of our readmissions were preventable since their causes rely on the transitional and outpatient quality of care after the initial hospitalization, including secondary prevention measures (24). On the other hand, as explained by Bjerkreim et al. (20), severe stroke patients, even when appropriately treated, are prone to infections due to repeated pulmonary aspiration and urinary catheterizations, and because strokes may affect the immunological status (20, 35). Furthermore, the natural disease history and the stroke early phase prothrombotic state may explain some readmissions due to vascular events, despite proper secondary prevention measures (20, 27).

Most stroke-related readmissions also occurred shortly after FELS-IH and persisted over the year. This finding is coherent with the aforementioned temporal pattern of readmission causes and highlights the need for more targeted specific interventions (34).

Hypertension, AF, and congestive heart failure are well-recognized stroke risk factors (27, 29) and were identified in our cohort univariate analysis as independent readmission/death risk factors. Thus, in order to prevent subsequent readmissions/deaths, besides the constant monitoring of stroke secondary prevention treatments, patients must also be aware of stroke warning signs and educated about the importance of controlling stroke risk factors and adhering to the recommended medication and behavioral changes in the long term (29, 36–39). This last goal may be achieved in the aftercare of stroke with well-designed and targeted multifactorial intervention programs of support, as described in the INSPiRE-TMS study (38).

Although smoking habits are considered a deleterious stroke risk factor (40), the univariate analysis showed that being a current smoker may be a protective factor of readmission/death. As explained elsewhere (41), this may be due to potential misreporting of patients' smoking status, or a bias since the sickest individuals (most prone to readmission/death) might had already stopped smoking because of their morbidity status.

Our study's multivariate analysis reinforced the evidence that age, previous and post-stroke functional status, and FELS-IH LoS are important independent factors of readmission/death (1, 5).

Although seldom referred in the literature (1, 4), the male sex was also an independent predictor of readmission/death in our study; this might be due to their proneness to recurrent IS (42); nevertheless, this hypothesis is not consensual in the literature (43).

Our study has some limitations. Since information about readmissions/death was collected retrospectively 3 months after FELS, there is an inherent data collection bias, which we mitigated using information from the medical records instead of only from administrative data. Also, our definitions for the vascular risk factors may have led to a decreased report rate, e.g., patients with no information regarding their smoking habits were considered non-smokers. We might have underestimated the true proportion of readmissions by not collecting data from private hospitals, but this information was probably registered posteriorly in the patients' NHS medical records, which we analyzed. Last, we did not include some complications in the FELS-IH or the type of discharge destination, which in other studies were linked to the readmission risk (1).

## Conclusion

Almost one-third of FELS survivors were readmitted/dead 1 year after their FELS-IH. This outcome persisted after the first months after stroke hospitalization in all stroke subtypes. More than half of readmissions were considered potentially avoidable or stroke related, and the main cause of potentially avoidable readmissions was continuation/recurrence of the event responsible for the initial admission or a closely related condition. Identifying potentially modifiable causes of readmissions and stroke survivors more prone to readmissions, as we have done in this study, may help organizations allocate resources and implement targeted readmission reduction policies.

## Data Availability Statement

The data that support the findings of this study is available from the population-based register (ACIN2) which is managed by its main investigators. The dataset used and analyzed in this study are available from the corresponding author on reasonable request and with permission of the ACIN2 investigators.

## Ethics Statement

This study was conducted in accordance with the World Medical Association Declaration of Helsinki and with the approval of the Ethics Committee of the Centro Hospitalar Universitário de São João and the Centro Hospitalar Universitário do Porto where it was performed.

## Author Contributions

PA was responsible for the study conceptualization, data acquisition, part of the statistical analysis and table elaboration, and drafting of the main manuscript. RM was the main responsible for the statistical analyses and table and figures elaboration, contributed to the study conceptualization/methodology, data acquisition, and article draft/review. DB contributed to the data acquisition and study conceptualization. EA, MS, and MC critically reviewed this article and contributed to the study conceptualization/methodology and article draft.

## Conflict of Interest

The authors declare that the research was conducted in the absence of any commercial or financial relationships that could be construed as a potential conflict of interest.

## Abbreviations

FELS: first-ever-in-a-lifetime stroke
FELS-IH: first-ever-in-a-lifetime stroke index hospitalization
NHS: National Health Service
TIA: transient ischemic attack
IS: ischemic stroke
ICH: intracerebral hemorrhage
SAH: subarachnoid hemorrhage
TOAST: Trial of Org 10172 in Acute Stroke Treatment
NIHSS: National Institutes of Health Stroke Scale
mRS: Modified Rankin Scale
ICD-9: International Classification of Diseases 9th Revision
PAR: potentially avoidable readmissions
HS: hemorrhagic stroke
LoS: length of stay
IQR: interquartile range
MI: myocardial infarction
SD: standard deviation.
